# Integration of Cell-Free Expression and Solid-State NMR to Investigate the Dynamic Properties of Different Sites of the Growth Hormone Secretagogue Receptor

**DOI:** 10.3389/fphar.2020.562113

**Published:** 2020-10-29

**Authors:** Emelyne M. Pacull, Franziska Sendker, Frank Bernhard, Holger A. Scheidt, Peter Schmidt, Daniel Huster, Ulrike Krug

**Affiliations:** ^1^ Institute for Medical Physics and Biophysics, University of Leipzig, Leipzig, Germany; ^2^ Institute of Biophysical Chemistry, Goethe University Frankfurt, Frankfurt am Main, Germany; ^3^ Center for Biomolecular Magnetic Resonance, Goethe University Frankfurt, Frankfurt am Main, Germany

**Keywords:** cell-free expression, DIPSHIFT, dynamics, G protein-coupled receptors, magic angle spinning, NMR

## Abstract

Cell-free expression represents an attractive method to produce large quantities of selectively labeled protein for NMR applications. Here, cell-free expression was used to label specific regions of the growth hormone secretagogue receptor (GHSR) with NMR-active isotopes. The GHSR is a member of the class A family of G protein-coupled receptors. A cell-free expression system was established to produce the GHSR in the precipitated form. The solubilized receptor was refolded *in vitro* and reconstituted into DMPC lipid membranes. Methionines, arginines, and histidines were chosen for ^13^C-labeling as they are representative for the transmembrane domains, the loops and flanking regions of the transmembrane α-helices, and the C-terminus of the receptor, respectively. The dynamics of the isotopically labeled residues was characterized by solid-state NMR measuring motionally averaged ^1^H-^13^C dipolar couplings, which were converted into molecular order parameters. Separated local field DIPSHIFT experiments under magic-angle spinning conditions using either varying cross polarization contact times or direct excitation provided order parameters for these residues showing that the C-terminus was the segment with the highest motional amplitude. The loop regions and helix ends as well as the transmembrane regions of the GHSR represent relatively rigid segments in the overall very flexible receptor molecule. Although no site resolution could be achieved in the experiments, the previously reported highly dynamic character of the receptor concluded from uniformly ^13^C labeled receptor samples could be further specified by this segmental labeling approach, leading to a more diversified understanding of the receptor dynamics under equilibrium conditions.

## Introduction

The growth hormone secretagogue receptor (GHSR) is one of more than 800 G protein-coupled receptors (GPCRs) in the human genome. In the last decade, there has been outstanding progress in the determination of crystal and cryo-EM structures of GPCRs mostly in the apo form, but also in complex with ligands, and even with intracellular binding partners such as G proteins or arrestins (Venkatakrishnan et al., 2013; Hilger et al., 2018; Shimada et al., 2018; Huang et al., 2020; Staus et al., 2020). These breakthroughs combined with the fact that at least 30% of the currently available drugs target these receptors (Hopkins and Groom, 2002; Garland, 2013) have pushed structure-based investigation of GPCR biology to the center of many current research activities. GPCR structural biology is fundamental for our general understanding of the mode of action of these molecules as well as for pharmacological intervention. But not only structural insights are essential for understanding GPCR function as these receptors are pronounced to be highly dynamic. Spectroscopic tools that allow for the investigation of the molecular dynamics of GPCRs have received increasing importance in the field (Kim et al., 2013; Solt et al., 2017; Shimada et al., 2018; Sušac et al., 2018). These methods typically require labeling with either fluorescence moieties (Tian et al., 2017), spin probes (Farrens, 2010), or NMR-active isotopes (Casiraghi et al., 2018; Franke et al., 2018).

For the investigation of GPCR dynamics *in vitro*, relatively large amounts of protein are required using expression in *E. coli* (Das et al., 2015; Schmidt et al., 2018), yeast (Eddy et al., 2018), mammalian (Ahuja et al., 2009) or insect cells (Isogai et al., 2016) as well as by cell-free (CF) expression techniques (Henrich et al., 2015; Krug et al., 2020). CF expression represents an interesting alternative to the traditional *E. coli* culture. Due to the open nature of the CF expression system, it can be optimized to suit specific requirements of the target protein. The reaction takes place either in a one compartment batch reaction or under continuous exchange with two compartments separated by a semipermeable membrane. Three expression reaction modes for membrane proteins are commonly used: 1) expression in the precipitated form, 2) expression in the presence of detergent micelles and 3) expression into lipid bilayers and nanodiscs (Schwarz et al., 2008; Rues et al., 2016). CF expression is especially valuable to selectively introduce isotopically labeled amino acid with a much lower scrambling rate, i.e., a reduced metabolic conversion between amino acids (Klammt et al., 2004; Bernhard and Tozawa, 2013).

**FIGURE 1:**
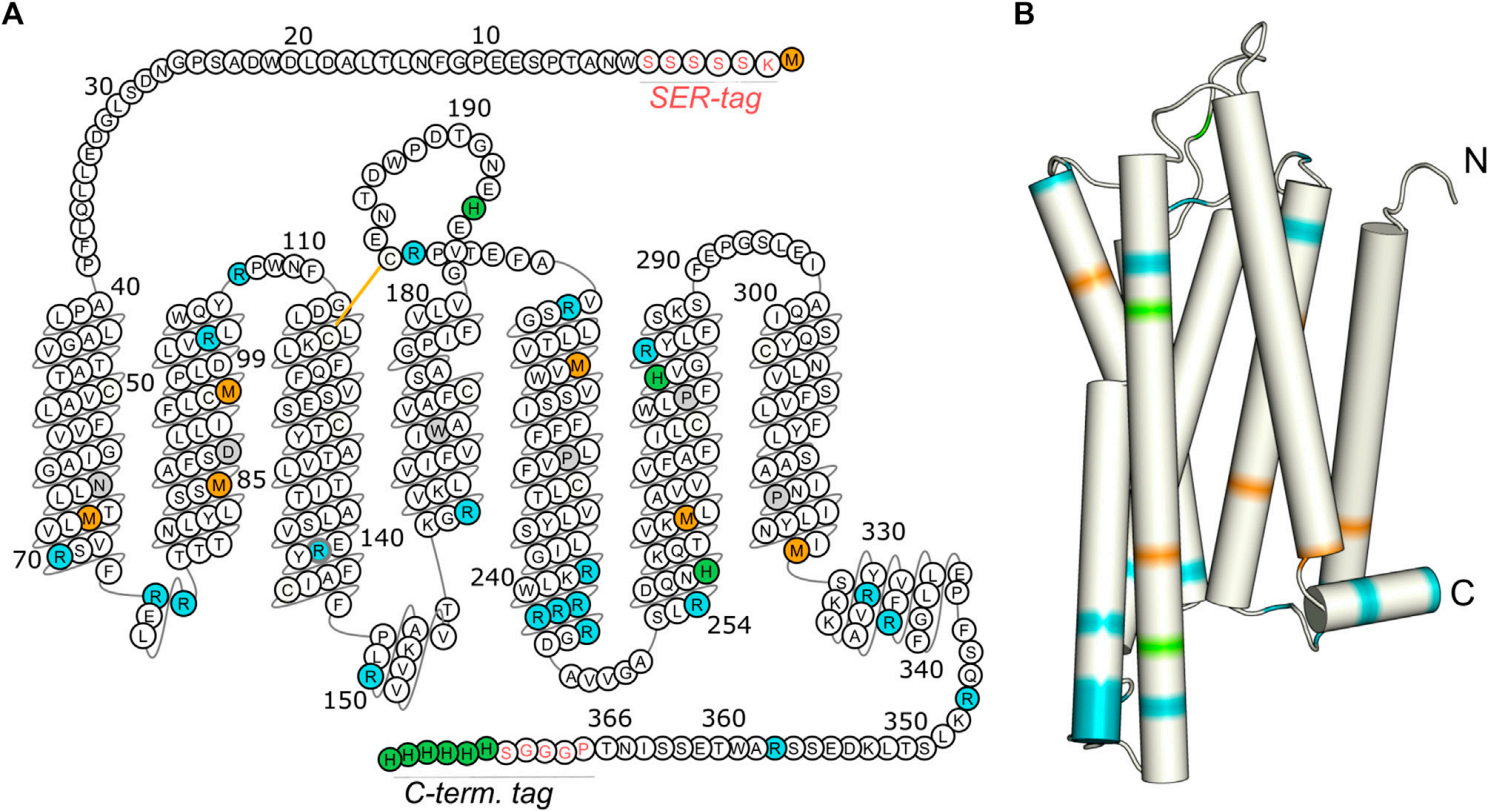
Isotopic labeling scheme for the characterization of the molecular dynamics of the transmembrane domain, the loop regions, and the C-terminus of GHSR. Methionines (orange) are mostly located in the transmembrane region, arginines (blue) are predominantly found at the ends of transmembrane helices and in loops, and histidines (green) are concentrated within the C-terminal His tag. The snake plot in **(A)** shows the amino acid sequence of the GHSR construct used for CF expression with the SER-tag at the N-terminus as well as the artificial sequence PGGGS and the His-tag at the C-terminus. Amino acids are numbered according to the native sequence of GHSR. Conserved amino acids are indicated in gray. The arrangement of the amino acids in the snake plot is given as in the GPCR database (Pándy-Szekeres et al., 2018). Panel **(B)** illustrates the structural model of GHSR (Bender et al., 2019). Full termini have not been modeled.

In the current work, CF expression was used to selectively label three amino acid types of GHSR with ^13^C (Figure 1). GHSR is a rhodopsin-like class A GPCR with the endogenous ligand ghrelin. This 28 amino acid long peptide is mainly known for its role in food intake and stimulation of appetite, but is also involved in growth hormone secretion and the modulation of behavior and mood (van der Lely et al., 2004; Kojima and Kangawa, 2005; Dickson et al., 2011). For a deeper understanding into ligand binding, the membrane-bound as well as the receptor-bound structures of ghrelin were described recently (Vortmeier et al., 2015; Bender et al., 2019). Furthermore, a first crystal structure was obtained for an antagonist bound GHSR (Shiimura et al., 2020). But GHSR signaling is not exclusively dependent on the presence of a ligand. The receptor shows a high constitutive activity of about 50% that has physiological importance as reflected in the fact that the loss of constitutive activity comes along with a short-stature phenotype and obesity in patients (Holst et al., 2004; Wang et al., 2004; Pantel et al., 2006; Damian et al., 2012). Also, *in vivo* formation of homo- as well as heterodimers likely serves in a potential mechanism for fine-tuning GHSR-mediated activity related to appetite regulation and food reward and affects intracellular responses but also the constitutive activity (Jiang et al., 2006; Chow et al., 2008; Rediger et al., 2011; Damian et al., 2015; Damian et al., 2018).

GHSR is involved in G protein signaling and β-arrestin recruitment. These pathways can be biased in distinct directions by mutations of the receptor’s amino acid sequence (Reiter et al., 2012; Shukla et al., 2013; Evron et al., 2014; Shukla et al., 2014). Ligands can also play an important role in biasing the receptor’s response (Klein Herenbrink et al., 2016; Kenakin, 2019).

Recent fundamental discoveries have established that GPCRs adopt multiple conformational states that are linked to distinct functional outcomes (Latorraca et al., 2017; Bostock et al., 2019). An ideal spectroscopic tool to characterize the dynamics of GPCRs is NMR spectroscopy (Bostock et al., 2019). In particular solid-state NMR spectroscopy under magic angle spinning (MAS) conditions allows investigation of membrane proteins in their natural membrane environment, in physiological buffer and at biological temperature (Huster and Madhu, 2014). Previous investigations focused on the description of the average dynamics of uniformly ^13^C labeled GHSR without any site resolution (Schrottke et al., 2017). In the current work, we extend our former studies and characterize the dynamics of specific regions of the molecule by specifically labeling amino acids that cluster in distinct regions of the receptor, i.e. the transmembrane domains, the loops, and the C-terminus. We describe the equilibrium dynamics of specific receptor regions using NMR-derived order parameters, which provide a measure for the amplitude of segmental motions of H-C bond vectors of three residue types of GHSR on a timescale faster than some tens of µs.

## Materials and Methods

### Preparation of Plasmid DNA

For CF expression of GHSR, the pIVEX2.3d vector (biotechrabbit GmbH, Hennigsdorf, Germany) was used. To avoid translation of the protein starting at the ATG codon of the *Nco*I site of this plasmid, ATG was mutated to a GCG codon using the QuikChange method from Stratagene and the PfuUltra II fusion HS DNA polymerase (Agilent, Waldbronn, Germany). The cDNA coding for GHSR was subcloned via *Nde*I and *Xma*I (NEB, Frankfurt, Germany) sites into the modified pIVEX2.3d vector using a standard PCR protocol as well as Phusion^®^ High Fidelity DNA Polymerase (NEB, Frankfurt, Germany), the forward primer 5’-CGA TAC **CAT ATG** AAA TCA TCA TCA TCA TCA TGG AAC GCG ACG CCC AGC-3’ and the reverse primer 5’-CGT ATA **CCC GGG** TGT ATT AAT ACT AGA TTC-3’ (each with the restriction sites in bold letters and the annealing part underlined; purchased from Life Technologies, Darmstadt, Germany). Constructs were validated by sequencing. Large amounts of plasmid DNA with concentrations between 600 and 1000 ng/µl were produced using the QIAGEN Plasmid Midi Kit (Qiagen, Hilden, Germany).

The expressed proteins consisted of the native amino acid sequence with the N-terminal tag inserted between the start methionine and the native residue Trp_2_ as well as an additional PGGGS sequence and a hexahistidine tag at the C-terminus (Figure 1A).

### *E. coli* Extract Preparation

For all CF experiments, S12 extracts were used. The preparation was modified from different protocols (Liu et al., 2005; Kim et al., 2006; Schwarz et al., 2007; Pedersen et al., 2011). To prepare the extract, *E. coli* Rosetta (DE3) cells were cultivated in 2× YTPG medium at 37°C and then grown in a fermenter containing 3 L of medium until the cells reached an optical density of about 4. Additives for growth of the cells in the fermenter are given in the Supplementary Table S1. Cells were harvested and resuspended in 10 mM Tris acetate (pH 8.2), 14 mM magnesium acetate, and 60 mM potassium acetate. Just before cell lysis, one tablet of cOmplete EDTA free (Roche Diagnostics, Mannheim, Germany) and 1 mM DTT were added per 50 ml of resuspended cells. The cells were lyzed using an APV 2,000 homogenizer and the solution centrifuged twice at 12,000 × *g* for 30 min. The supernatant was incubated with 400 mM NaCl solution at 42°C for 45 min. Then, the turbid extract was dialyzed at 4°C in two steps in 10 mM Tris acetate (pH 8.2), 14 mM magnesium acetate, 60 mM potassium acetate and 0.5 mM DTT. After a final centrifugation step at 12,000 × *g* and 4°C for 30 min aliquots were snap frozen. For each preparation of S12 extract, the optimal Mg^2+^ concentration was determined in a range of 14–20 mM magnesium acetate. Additionally, a second set of S12 extract, containing T7 polymerase was prepared. The same procedure was followed but T7 expression was induced with IPTG at OD_600_ = 1 during cultivation of the cells in the fermenter.

### Preparations for Cell-Free Expression

The protocol for CF expression was modified from (Schwarz et al., 2007) and solutions were prepared accordingly. Amino acids (Sigma Aldrich, Taufkirchen, Germany) were dissolved in 100 mM Hepes, pH 7.4, or ddH_2_O to a concentration of 20 mM for Tyr, 50 mM for Trp and 100 mM for all other amino acids. The pH was adjusted to a value between 7.0 and 7.4. The amino acids were combined in an amino acid mix with a final concentration of 5 mM for each amino acid, in an RCWMDE mix with a final concentration of 8.4 mM for Trp and 16.7 mM for the other five and in an AFLSTV mix with a final concentration of 16.7 mM of each amino acid. The RCWMDE and the AFLSTV mixes provided an additional amount of the most “problematic” amino acids and of the six most abundant amino acids of the protein sequence, respectively. During the experiments, it turned out to be advantageous to add Cys separately. For selective amino acid labeling, the natural amino acids were replaced by ^13^C-labeled or ^13^C/^15^N-labeled amino acids that were purchased from Sigma Aldrich or Eurisotop (Saarbrücken, Germany). Lithium acetyl phosphate (Sigma Aldrich) was dissolved in ddH_2_O to a concentration of 1 M. The reagent was only completely dissolved after incubation of the slurry solution at 30°C overnight and subsequent freezing at -20°C.

### Cell-Free Expression

The reaction mix (RM) and feeding mix (FM) of the CF expression were pipetted according to the scheme shown in the supplement (Supplementary Table S2). All solutions were stored on ice while pipetting the mixes of the CF expression. Folic acid was prepared fresh and protected from light. For each experiment, a fresh 50× cOmplete solution (EDTA-free Protease Inhibitor Cocktail) was prepared. S12 extract was added to the RM with a final concentration of 40% (v/v). RiboLock RNAse inhibitor and T7 RNA Polymerase were purchased from Life Technologies (Darmstadt, Germany). Alternatively, S12 extract already containing T7 polymerase was used. CF expression was carried out with the RM filled in a dialysis bag (MWCO 12,000–14,000 Da, ZelluTrans/Roth, Karlsruhe, Germany) that was placed in a weighing dish filled with the FM. The weighing dish was covered by a second one and parafilm sealed the reaction chamber. The reaction was incubated 24 h at 34°C and 100–150 rpm.

### Solubilization, Purification and Reconstitution of Growth Hormone Secretagogue Receptor

GHSR was expressed as precipitate in the dialysis bag. The expression was terminated after 24 h and the precipitate solubilized by addition of 9 ml of 50 mM sodium phosphate buffer (pH 6.5), 15 mM SDS and 50 mM DTT per 1 ml RM. Further refolding steps were carried out as described before (Schmidt et al., 2018). Briefly, DTT was removed by dialysis in two steps against a buffer containing 50 mM sodium phosphate buffer (pH 6.5) and 15 mM SDS. After dialysis the pH was adjusted to pH 8 and the denatured protein subjected to immobilized metal affinity chromatography using a 5 ml HisTrap™ HP column (GE Healthcare, Germany). The protein was eluted by a pH shift. Protein purity was checked by SDS-PAGE analysis and concentration determined using a NanoDrop Spectrophotometer (Thermo Scientific). The protein was stored at -20°C or directly reconstituted in lipid membranes for immediate use. For reconstitution, the disulfide bridge was formed during dialysis under addition of the glutathione redox system (2 mM reduced glutathione, 1 mM oxidized glutathione) and by reduction of the SDS concentration from 15 to 2 mM. Then, the receptor was incorporated into small lipid bicelles of 1,2-dimyristol-*sn*-glycerol-3-phosphocholine (DMPC, chain-deuterated DMPC-*d*_54_ or headgroup deuterated DMPC-*d*_13_) and 1,2-diheptanoyl-*sn*-glycero-3-phosphocholine (DHPC) (molar ratio of 1:200:800, receptor:DMPC:DHPC). All lipids were purchased from Avanti Polar Lipids, Inc. (Alabaster, United States) and used without further purification. Finally, residual detergent was removed in multiple incubation steps with 50 mg/ml BioBeads SM2 (Bio-Rad, München, Germany) increasing the q-value from 0.25 to above 10 representing essentially DMPC lipid bilayers with traces of DHPC detergent left.

### Ligand Binding Assay

The activity of the expressed and refolded GHSR was demonstrated by ligand binding (Schrottke et al., 2017). To this end, the receptor was reconstituted in DMPC/DHPC bicelles with a molar ratio of 1:600:2400 (receptor:DMPC:DHPC) corresponding to a q value of 0.25. The binding of ATTO520-labeled ghrelin to the GHSR prepared by CF expression was measured using the previously described fluorescence polarization assay (Schmidt et al., 2018; Bender et al., 2019). Experiments were performed in a 10 mm quartz cuvette on a FluoroMax-2 (JOBIN YVON) at 20°C with linear polarized light, an excitation wavelength of 500 nm, an emission wavelength of 540 nm, and 90° detection angle. Three independent measurements were carried out in duplicate or triplicate. Saturation binding data were fit with a sigmoidal dose-response curve using the Origin software. To assess the binding of ATTO520-ghrelin to the membrane alone, the measurements were repeated with empty bicelles (two measurements in triplicate).

### NMR Spectroscopy

Between 1.5 and 3 mg of isotopically labeled GHSR were reconstituted in DMPC, DMPC-*d*_54_ or DMPC-*d*_13_ membranes. In two additional centrifugation steps, excess water was removed using gravitational forces of 21,500 × *g*. Subsequently, samples were frozen in liquid nitrogen and filled into 3.2 mm MAS rotors. ^13^C MAS NMR experiments were carried out on Bruker Avance III 600 MHz or Avance Neo 700 MHz NMR spectrometers using triple resonance MAS probes with a 3.2 mm spinning module at a temperature of 37°C. The ^1^H-^13^C dipolar couplings were measured using the DIPSHIFT pulse sequence (Munowitz et al., 1981) at an MAS frequency of 5 kHz with the 90° pulses adjusted to 4 μs for both channels. Either direct excitation or cross polarization (CP) with contact times of 20, 700, or 2000 µs was used for the excitation of the ^13^C nuclei. For the short contact time of 20 µs, the RODEO DIPSHIFT pulse sequence was used to minimize the distortions in the dephasing curves (Kurz et al., 2013). For heteronuclear decoupling, the spinal sequence (Paëpe et al., 2003) with an rf field strength of 65 kHz was used. The FSLG sequence (Bielecki et al., 1989) with an rf field of 80 kHz was used for homonuclear ^1^H-^1^H decoupling. The order parameters were calculated as the ratio of the motionally averaged dipolar coupling strength and the rigid limit value of the dipolar coupling (Huster et al., 2001). The dipolar coupling strength was derived from best fit of numerically simulated dipolar dephasing curves fitted to the experimental dephasing curves measured over one rotor period (using seven or nine increments). To check the influence of the lipid signals, DIPSHIFT experiments were repeated with a sample of DMPC/DHPC membranes without receptor.

Dipolar Assisted Rotational Resonance (DARR) experiments (Takegoshi et al., 2001; Takegoshi et al., 2003) were carried out to disperse the NMR signals in two dimensions utilizing ^13^C-^13^C correlations within the labeled amino acids of GHSR. From these spectra, chemical shift regions to be analyzed were defined. For each labeled amino acid type, the DARR NMR spectrum was recorded at 11,777 Hz MAS frequency and at a temperature of -30°C, using a mixing time of 10 ms and a CP contact time of 2 ms.

Static ^31^P NMR spectra of the multilamellar vesicles were acquired on a Bruker Avance III 600 NMR spectrometer using a Hahn-echo pulse. A ^31^P 90° pulse length of 10 µs, a Hahn-echo delay of 30 µs, a spectral width of 50 kHz, and a recycle delay of 3 s were used. Continuous-wave proton decoupling was applied during signal acquisition.

## Results

### Cell-Free Expression of Growth Hormone Secretagogue Receptor

For CF expression of the GHSR, the encoding DNA was inserted into the pIVEX2.3d vector (Rogé and Betton, 2005) with an AT-rich sequence at the 5’ end (Haberstock et al., 2012). The maximum expression yield of about 1.4 mg per 1 ml reaction volume was obtained for the construct with the N-terminal SER-tag (sequence: KSSSSS, Figure 1), in comparison to 0.7 mg for the H-tag (sequence: KPYDGP), and 0.9 mg for the AT-tag (sequence: KYYKYY). A fairly simple experimental setup was chosen for CF expression with the reaction chamber built from weighing dishes containing the dialysis bag. Expression was optimized to yield highest amounts of protein at 34°C.

### Troubleshooting in Cell-Free Expression

Due to the large number of components required for CF expression, even small changes in the procedure can result in very low expression yields. The most critical aspects for this study are summarized. i) T7 RNA polymerase was very sensitive to temperature alterations. In our hands, any interruption of the cold chain led to a substantial loss of polymerase activity. Therefore, S12 extract containing T7 polymerase was prepared and successfully used for the CF expression of the latest samples. ii) Dissolving phosphoenolpyruvate (PEP) in water by adjusting the pH under titration of potassium hydroxide was very sensitive. At pH > 7, the unstable phosphate group of PEP was hydrolyzed. This most likely led to a reduction in the concentration of free Mg^2+^ ions and thereby inhibition of the CF expression (Kim and Swartz, 1999). iii) After a few freeze/thaw cycles, we observed a precipitate and perceived a considerable smell of the stock solution of 100 mM cysteine, likely resulting from oxidation of cysteine to cystine. Also, the RCWMDE mix became turbid due to the precipitation of cystine. This lowered expression yields significantly. Thus, cysteine was not added as part of the amino acid mixes, but separately from a freshly dissolved, clear stock solution.

### Purification and Reconstitution of Growth Hormone Secretagogue Receptor

**FIGURE 2:**
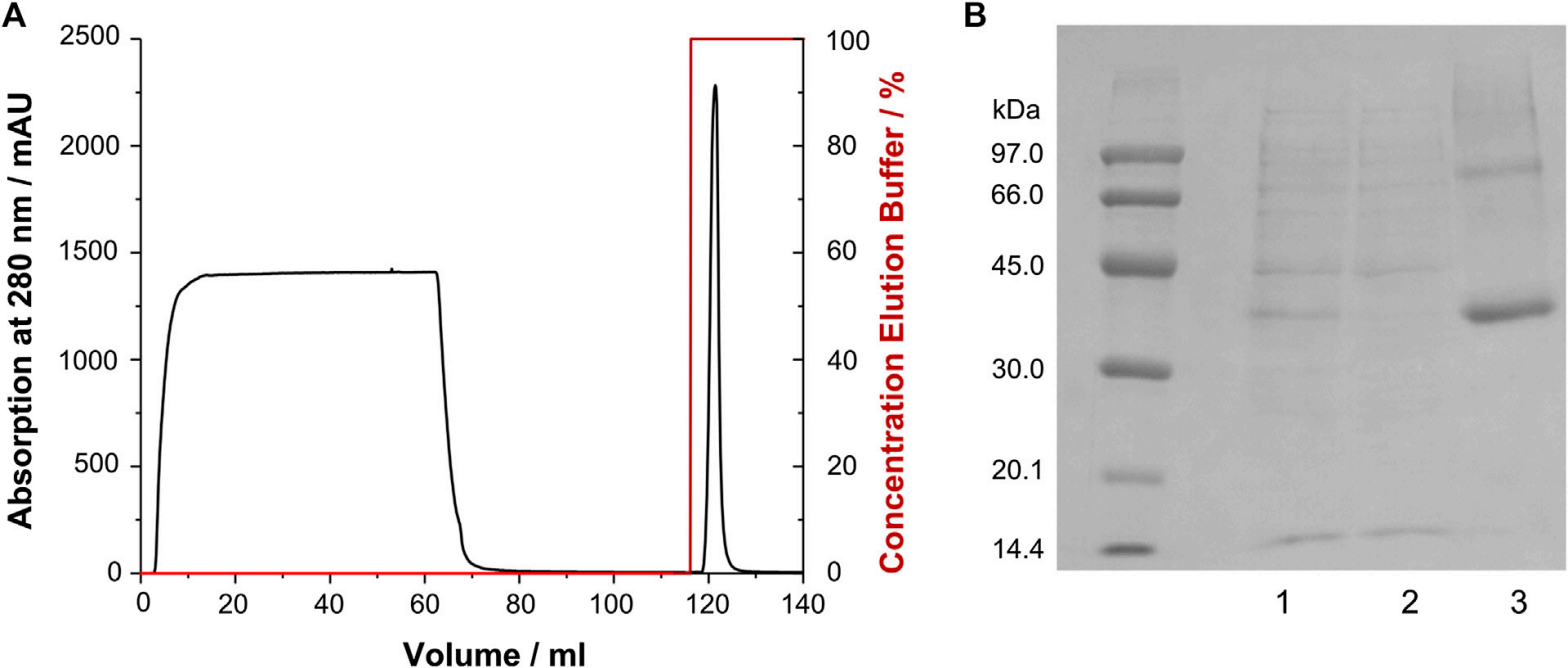
Purification of GHSR (labeled with ^13^C/^15^N methionine) from CF expression. **(A)** Immobilized metal affinity chromatogram of GHSR, which was purified via its C-terminal hexahistidine tag. The protein was eluted by a pH shift. **(B)** SDS-PAGE analysis of the purification of GHSR (molecular weight 43 kDa) showing 1) the solubilized precipitate, 2) the flow through of the chromatography step, and 3) purified GHSR.

CF expression of soluble GPCRs can be cumbersome and required careful optimization, e.g., regarding the type and composition of lipid mixes and detergents added to the solution. Furthermore, stability of those preparations is sometimes limited. Therefore, we adopted previously established protocols from recombinant *E. coli* expression (Schmidt et al., 2009; Schmidt et al., 2010; Schmidt et al., 2018) to the treatment of receptor aggregates obtained from CF expression. The GHSR was expressed as insoluble precipitate, which was treated similar as the inclusion bodies obtained from *E. coli* fermentation. The precipitate was solubilized by SDS and DTT, purified via immobilized metal affinity chromatography (Figure 2) and reconstituted by *in vitro* folding. To this end, first the disulfide bridge was formed, second the GHSR was incubated in DMPC/DHPC bicelles (q = 0.25), and finally planar membranes were formed by removal of most of the DHPC. The refolding yield was about 40–50%. For NMR spectroscopy, either non-deuterated DMPC, chain deuterated DMPC-*d*_54_ or lipid head group deuterated DMPC-*d*_13_ was used. Static ^31^P NMR experiments (spectra shown in Supplementary Figure S1) confirmed the bilayer structure of our DMPC preparations. In the presence of GHSR, the chemical shift anisotropy of the ^31^P NMR signal decreased as an indication for increased headgroup orientation and/or mobility in response to receptor interaction as first reported by (Scherer and Seelig, 1989). Furthermore, we observed an increase in the isotropic lipid signal from 5% in the absence to 19% in the presence of GHSR (Supplementary Figure S1).

Ligand binding of reconstituted GHSR was shown by a fluorescence polarization assay. In three independent experiments, an average EC_50_ of 56 ± 30 nM was determined (Supplementary Figure S2).

### Isotope Labeling Strategy and Measurement of ^13^C-^13^C Correlations to Study Specific Regions of the Growth Hormone Secretagogue Receptor

**FIGURE 3:**
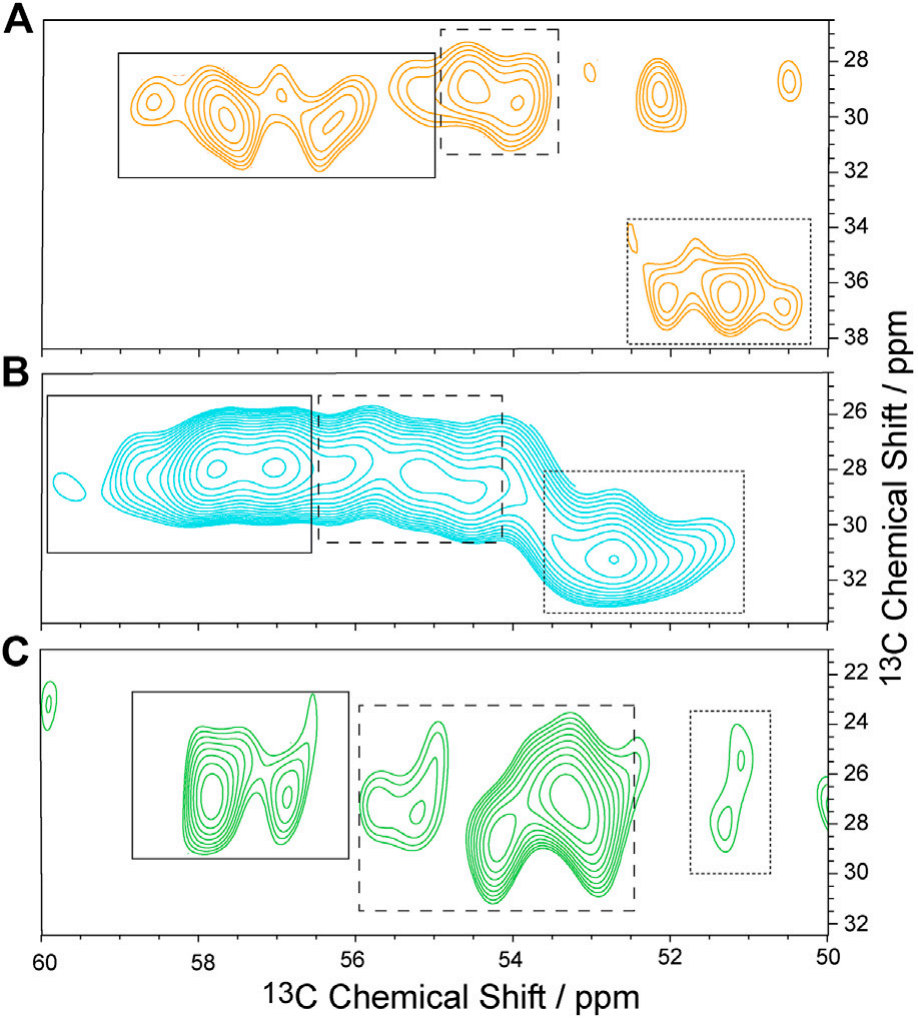
Sections of ^13^C-^13^C DARR NMR spectra of isotopically labeled GHSR in membranes. Experiments were carried out at -30°C and an MAS rate of 11,777 Hz. GHSR was labeled with ^13^C/^15^N-Met **(A)**, ^13^C/^15^N-Arg **(B)**, and ^13^C/^15^N-His **(C)**. Only Cα/Cβ correlations are shown. Solid, dashed, and dotted boxes indicate the spectral areas analyzed for signals with α-helical, random coil, and β-sheet secondary structures, respectively. Full ^13^C-^13^C DARR NMR spectra along with 1D cross sections are provided in **Supplementary Figure S3**.

To characterize the dynamics of individual segments of the GHSR, amino acids clustered in specific regions of the receptor were isotopically labeled. For an overview of the labeled target segments of GHSR, see Figure 1. Methionine (Met) was chosen as the amino acid to represent the transmembrane segments as six out of seven Met residues of the receptor are located in the core region of the molecule. The seventh Met is the start methionine on the N-terminus and putatively highly mobile. Using a ^13^C-^13^C DARR NMR experiment, the relevant chemical shift regions to be further analyzed were identified (see full ^13^C-^13^C DARR spectra in Supplementary Figure S3). In the DARR experiment, the polarization is transferred from ^1^H to the adjacent ^13^C nucleus and then to other ^13^C nuclei in close proximity to obtain inter residue correlations (Takegoshi et al., 2001; Takegoshi et al., 2003). The DARR spectrum of ^13^C-methionine labeled GHSR showed correlations between the Cα to the Cβ and Cγ atoms but not to Cε as a mixing time of 10 ms was not sufficient for the long-range magnetization transfer via the thiol ether. The majority of NMR signals in the Cα region is relatively well resolved and spans a chemical shift range from 53.0 to 59 ppm, which corresponds to α-helical and coil structure (Figure 3A). The spectral intensity of the cross peak around 51/36 ppm corresponds to the β-sheet region and indicates aggregated GHSR. The secondary structure assignment was based on the Cα-Cβ chemical shift difference in comparison to the literature (http://www.bmrb.wisc.edu/published/Ikura_cs_study/).

As representatives of putatively more flexible segments of the GHSR (see Figure 1), arginine (Arg) residues were isotopically labeled. According to the current structural model of GHSR (Bender et al., 2019) and the GPCR database (Pándy-Szekeres et al., 2018), the arginines cluster in the flanking regions of the α-helices and are well represented in the loops that connect these secondary structure elements. Due to limited resolution, it was not possible to resolve all 21 Arg of GHSR in the 2D NMR spectra (Figure 3B). The α-helical and random coil chemical shift range spreads from 54.5 to about 59.5 ppm, which covers most of the chemical shift range detected.

Histidine (His) residues were chosen to describe the dynamics of the C-terminal hexahistidine tag. Furthermore, GHSR contains only three additional His, His_186_ in the extracellular loop 2 as well as His_258_ and His_280_ in α-helix 6. For His, chemical shifts between 56 and 58.5 ppm indicate α-helical structures. Most intensity is found between 53 and 55 ppm, which refers to random coil structure and likely represents the signals from the histidine tag and the loop His_186_ and were assigned as such (Figure 3C).

### Determination of Segmental Order Parameters of Growth Hormone Secretagogue Receptor by DIPSHIFT Experiments

Investigation of the molecular dynamics of proteins by solid-state NMR is possible by measuring the motionally averaged ^1^H-^13^C dipolar couplings using separated local field NMR experiments carried out under MAS (Waugh, 1976). Here, the robust DIPSHIFT pulse sequence (Munowitz et al., 1981) was used for this purpose. As motions of a given ^1^H-^13^C bond vector with a certain amplitude scale down dipolar couplings, the magnitude of the measured dipolar coupling (*D*_exp_) contains information on the motional amplitude. It is convenient to calculate motional order parameters by dividing the measured motionally averaged dipolar coupling (*D*_exp_) by the rigid limit value (*D*_rigid_) when no motion is present to obtain the molecular order parameter according to *S* = *D*_exp_/*D*_rigid_. Thus, the order parameter ranges from 0 for isotropic mobility to 1 for complete rigidity. In our study, DIPSHIFT experiments were carried out at 37°C using varying CP contact times as well as direct polarization. As discussed before (Schmidt et al., 2014), polarization transfer by CP introduces a motional bias as the polarization transfer utilizes the dipolar interaction between the abundant spins with high gyromagnetic ratio (^1^H) to the rare spins with low gyromagnetic ratio (^13^C) to enhance sensitivity. As the magnitude of the dipolar coupling depends on the motional amplitude of a given bond vector, the efficiency of the polarization transfer is biased by motions. CP polarization transfer is very efficient for highly rigid sites but less efficient for more mobile and very inefficient for highly mobile sites. The length of the CP contact time is the parameter that can be used to polarize only rigid or also mobile sites. However, spectral intensity decreases with increasing CP contact time due to *T*_1ρ_ relaxation during application of the spin lock field. At a very short CP contact time, only highly rigid sites are polarized, while using longer CP contact times allows for efficient polarization of more mobile sites. Accordingly, if a short CP contact time is used, only the rigid segments of a molecule are detected, while longer CP contact times allow for detection of both rigid and mobile sites. If ^13^C NMR spectra are directly polarized, rigid and mobile sites are equally excited.

Here, we used DIPSHIFT experiments with varying CP contact times of 20, 700 and 2000 µs as well as direct polarization. In the latter case, all carbons (including those from the lipids) were excited equally. To determine the motionally averaged dipolar coupling constants, the dipolar dephasing curves were detected during *t*_1_ evolution and compared to numerical simulations using the integrals of initially defined spectral regions. The same integration ranges were used for all experiments of one type of labeled GHSR.

The GHSR was reconstituted into DMPC with a molar ratio of 1:200 (receptor to DMPC with traces of DHPC left). Thus, the ratio of lipid to receptor signals was relatively high which improves functionality of the receptor but provides low NMR signal. At such low protein concentration, care has to be taken to avoid detecting the influence of the mobile lipid signals on the NMR signals of the receptor backbone sites. The DMPC headgroup signals (α, β, and γ protons), which resonate within the protein backbone Cα region are very mobile and feature very small order parameters (*S* < 0.04) (Huster and Arnold, 1998), which leads to less than 1% dipolar dephasing in DIPSHIFT experiments at an MAS frequency of 5 kHz and can thus safely be neglected in the analysis of the GHSR motions. The lipid glycerol signals resonate at lower field (G-1,3 at 64 ppm and G-2 at 71 ppm, see Supplementary Figure S4) such that no signal overlap with Met, Arg, and His protein backbone sites has to be considered. Nevertheless, we prepared GHSR samples in DMPC, headgroup deuterated DMPC-*d*_13_ and chain deuterated DMPC-*d*_54_ but did not observe any systematic alteration in the order parameter suggesting any influence of lipid headgroup signals.

For the determination of the order parameters of the protein side chains, exclusively DMPC-*d*_54_ was used to filter out the contributions from the aliphatic lipid signals in the CP MAS NMR spectra for a relatively clean detection of the protein Hβ-Cβ dipolar couplings. However, care has to be applied as NMR signals from undeuterated residual DHPC and the C-2 methylene signal of DMPC-*d*_54_ which is only partially deuterated (information provided by the manufacturer) influence the measurement of the receptor side chain dipolar couplings. Also, in directly polarized DIPSHIFT spectra, an influence of mobile lipids on protein side chain order parameters cannot be excluded. For comparison, a sample of pure DMPC membranes without receptor was investigated under the same NMR conditions and DIPSHIFT order parameters of the lipid signals were determined with values of *S* < 0.2 for the chain segments and negligible order parameters for the lipid headgroups (Supplementary Table S3). An NMR spectrum of DMPC membranes with assignment of the lipid signals is given in the supplement (Supplementary Figure S4).

### Analysis of the Molecular Order Parameters of Methionine, Arginine, and Histidine Residues of Growth Hormone Secretagogue Receptor

**FIGURE 4:**
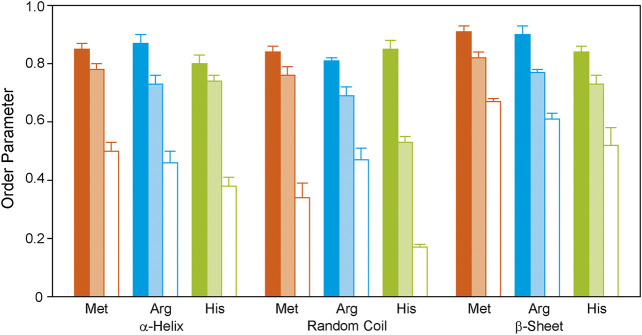
^1^H-^13^C DIPSHIFT order parameters of the backbone Cα atoms of isotopically labeled GHSR reconstituted in DMPC membranes determined at 37°C. Methionines were labeled to represent the transmembrane region (orange), arginine residues were labeled to provide information on the dynamics of the ends of the helices and loops (blue), and histidine residues report the dynamics of the C-terminus (green). Order parameters of NMR signals assigned to α-helical, random coil, and β-sheet secondary structures are shown as bars. Order parameters were determined from DIPSHIFT experiments using CP contact times of 20 µs (filled bars), and 700 µs (pale bars), as well as direct polarization (open bars). Error bars reflect the standard error of the mean. Exemplary DIPSHIFT spectra and dephasing curves used to calculate the order parameter are given in **Supplementary Figure S5**.

The methionine residues were ^13^C labeled to provide information on the dynamics of the helical segments of the receptor. Inspection of the distribution of the Met residues of GHSR (Figure 1) reveals, that five Met are located well within the α-helical region, Met_326_ is located at the end of helix seven before the loop to helix 8. The N-terminal Met_1_ is expected to be highly mobile and not significantly polarizable by CP. For analysis of the DIPSHIFT spectra, we grouped all NMR signals with α-helical chemical shift (55.0–59.2 ppm) as no assignments are available (Figure 3). Second, we analyzed the spectral region from 52.7 to 54.8 ppm (coil chemical shift range), likely to contain Met_326_ and Met_1_. Spectral intensity below 52.7 ppm was considered β-sheet stemming from aggregated receptor. All determined order parameters from DIPSHIFT experiments with varying CP contact times and direct excitation are reproduced in Supplementary Table S4. Representative DIPSHIFT spectra and dipolar dephasing curves are shown in Supplementary Figure S5. For illustration purposes, we plot the order parameters determined from a DIPSHIFT experiment with a very short CP contact time of 20 µs, the standard contact time for ^13^C NMR of 700 µs, and direct excitation in Figure 4. Clearly, very different order parameters are determined for each condition. At 20 µs CP contact time, only the highly rigid segments are excited, the contact time of 700 µs excites very rigid as well as moderately mobile sites, and in direct excitation, all sites are excited equally. From the comparison of the three conditions, conclusions about the heterogeneity of the distribution of motions in GHSR can be derived.

At 20 µs CP contact time, only the rigid Met residues likely coming from the central helix motifs are excited featuring high order parameters around 0.85 ± 0.02. This indicates that these sites undergo only small angle fluctuations. Order parameters decrease when DIPSHIFT experiments are carried out with a longer CP contact time as also more mobile segments are polarized (Figure 4). When all Met residues are equally excited by direct polarization, low order parameters of 0.50 ± 0.03 are observed for the helical Met residues and 0.34 ± 0.05 for Met_1_ and Met_326_ in coil conformation are measured, suggesting high dynamics and sizable motional amplitudes. The signal assigned to β-sheet likely from aggregated receptor accounting for 23% of the spectral intensity (Figure 3) is very rigid under both conditions with order parameters of 0.91 ± 0.02 and 0.67 ± 0.01, measured with CP with short contact time or direct polarization, respectively.

Out of the 21 arginine residues in the sequence of GHSR, twelve are localized at the transmembrane helix ends, two in helix 8, and seven in the loops and termini. We assigned chemical shifts between 56.7 and 60.2 ppm to the α-helical structures, chemical shift between 54.0 and 56.6 ppm to coil structure, and chemical shifts <53.3 ppm to β-sheet structure from aggregated receptor, accounting for 22% of the total signal intensity. These rigid sites again show very high order parameters of 0.87 ± 0.03 for the helical and to 0.81 ± 0.01 for the loop Arg residues determined in DIPSHIFT experiments with 20 µs CP contact times. The Arg residues of the aggregated receptor are expectedly very rigid (*S* = 0.90 ± 0.03). However, DIPSHIFT experiments with direct excitation detect much lower order parameters of 0.46 ± 0.04 for the helical Arg and 0.47 ± 0.04 for the loop structures agreeing with the high mobility also found for the Met sites. Similarly, the aggregated receptor is rather immobile under these conditions (*S* = 0.61 ± 0.02).

The His residues of GHSR cluster at the C-terminus of GHSR in form of the hexahistidine purification tag. Furthermore, the protein sequence of GHSR comprises two histidines located in transmembrane helix 6 and 1 in extracellular loop 2. We analyzed the α-helical spectral region (56–58.8 ppm) likely representing His_258_ and His_280_, the coil region (52.5–56 ppm) representing His_186_ and the signals from the histidine tag. As for the other two isotopic labels, the β-sheet region (<52.5 ppm) was assigned to aggregated receptor which accounted for 5% of the spectral intensity. DIPSHIFT experiments at a low CP contact time show similarly high order parameters around 0.8 which are similar for the helical and coil signals. However, it should be emphasized that only very little intensity was found for the His signal with random coil chemical shift at a CP contact time of 20 µs confirming that the His tag is very mobile. In the directly excited DIPSHIFT spectra, the α-helical sites likely representing His_258_ and His_280_ are very mobile (*S* = 0.41 ± 0.03). Highest mobility is observed for the coil signals assigned to His_186_ and the His tag featuring a very low order parameter of 0.17 ± 0.01.

**FIGURE 5:**
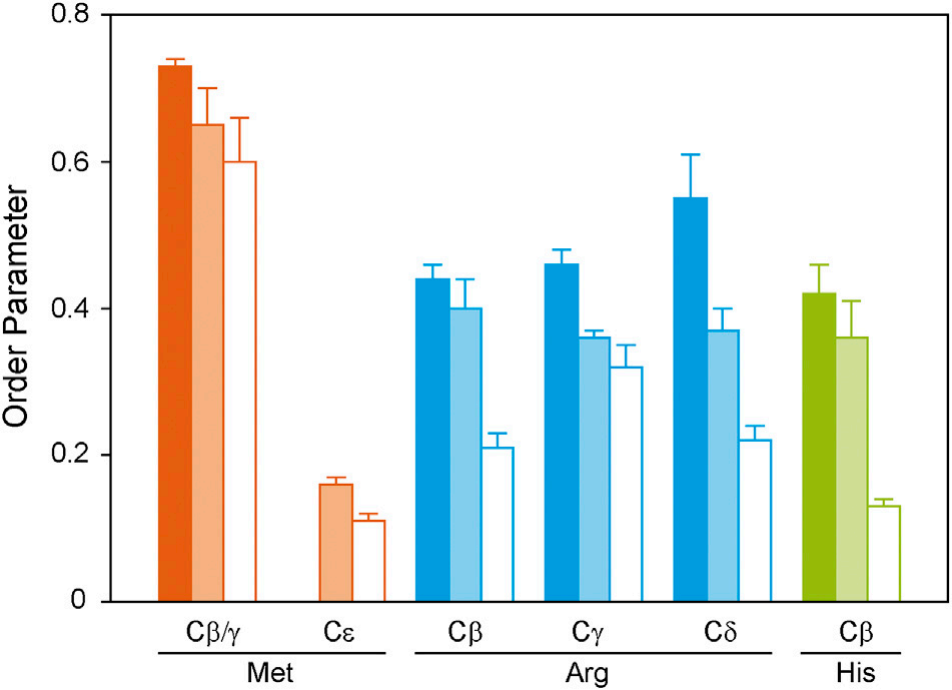
^1^H-^13^C DIPSHIFT order parameters of the side chain atoms of isotopically labeled GHSR reconstituted in DMPC-*d*_54_ membranes determined at 37°C. Methionines were labeled to represent the transmembrane region (orange), arginines were labeled to provide information on the dynamics of the ends of the helices and loops (blue), and histidines report the dynamics of the C-terminus (green). Order parameters were determined from DIPSHIFT experiments using cross polarization with a contact time of 20 µs (filled bars), 700 µs (pale bars), and direct polarization (open bars).

We also investigated the molecular mobility of the side chain sites of the Met, Arg and His residues of GHSR. To this end, we only analyzed the NMR spectra acquired in DMPC-*d*_54_ to avoid signal superposition with the highly mobile phospholipid chains. All determined side chain order parameters are shown in Supplementary Table S5 and illustrated in Figure 5 for data acquired using DIPSHIFT experiments excited by CP with 20 and 700 µs contact time or direct polarization.

Generally, in proteins side chain order parameters are lower than backbone order parameters (Reuther et al., 2006). Furthermore, if side chain order parameters are measured via DIPSHIFT experiments with a short CP contact time, they are much higher than for directly polarized DIPSHIFT experiments paralleling the situation encountered for the backbone. Due to poor resolution, no differentiation between secondary structure elements was possible for the side chain signals. Met side chains show the highest side chain order parameters for Cβ/Cγ, which could not be separated spectroscopically (Figure 5). The methyl group at Cε could not be polarized using CP with a short contact time. However, it shows a very low order parameter in the directly polarized DIPSHIFT experiments. For the Arg residues, side chain order parameters are also lower than for the backbone sites. Also, order parameters do not decrease as a function of side chain length, which could be related to the involvement of the guanidinium moiety in hydrogen bonds or salt bridges stabilizing the tertiary structure of GHSR. The His Cβ shows the lowest order parameters in agreement with the largest contribution from the mobile His tag on the C-terminus.

## Discussion

CF expression represents an excellent method for selective amino acid type labeling of proteins because of the open nature of the CF expression conditions and an overall low rate of isotopic scrambling (Hoffmann et al., 2018). Here, we established a fairly simple protocol that allowed expression of GHSR, a typical representative of class A GPCRs, in the precipitated form. In combination with already established procedures for GPCR purification and reconstitution, based on the preparation of functional receptor from *E. coli* inclusion bodies (Schmidt et al., 2018), GHSR was obtained in relatively high yields and after reconstitution into planar lipid membranes with sufficient stability for solid-state NMR experiments. Alternatively, CF expression of GPCRs in the presence of detergents or nanodiscs directly in the reaction mix has been reported which may promote spontaneous insertion of the newly synthesized receptors into these membrane mimetics (Rues et al., 2016). Although this strategy does not require additional reconstitution steps, CF expression in the presence membrane mimetics often suffers from low stability of the sample, missing functionality or just low yields, which increase the difficulties for the subsequent NMR experiments. Our protocol allowed the reconstitution of >80% functional receptor into DMPC membranes leaving about 20% protein aggregates, which could be identified according to their β-sheet chemical shift. 2D integration of the crosspeak volumes in the DARR spectra carried out for the cross peaks with α-helical, coil and β-sheet chemical shifts allowed quantification of the relative proportion of aggregated GHSR. Due to the spectral separation, the NMR signals of the aggregated receptor do not interfere with the motional analysis of the correctly folded GHSR (see Figure 3).

Although great for simplifying NMR spectra, residue type labeling provokes the difficulty of not obtaining sequential assignments as only isolated amino acids are labeled and sequential connectivities are mostly absent. Usually, the NMR assignments can only be obtained in extensive and tedious site-directed mutagenesis approaches (Isogai et al., 2016; Imai et al., 2020). An additional difficulty in residue type specific labeling of GPCRs arises from the structural heterogeneity and intermediate timescale dynamics of these molecules, which decreases NMR spectral resolution. In contrast, other heptahelical membrane proteins such as bacterial rhodopsins feature much better structural homogeneity allowing for site-specific assignments and analysis of molecular dynamics (Saitô et al., 2000; Barré et al., 2003; Yang et al., 2011; Good et al., 2014).

Previous work has shown that in addition to the dynamic conformational changes that GPCRs undergo during activation (Manglik et al., 2015; Isogai et al., 2016; Imai et al., 2020), these receptors are in general very dynamic membrane proteins even under equilibrium conditions in liquid crystalline membranes (Schmidt et al., 2014; Thomas et al., 2015). This was demonstrated by solid-state MAS NMR spectroscopy showing that fully ^13^C-labeled GHSR (Schrottke et al., 2017) and neuropeptide Y receptors (Schmidt et al., 2014; Thomas et al., 2015) feature low molecular order parameters of ∼0.5–0.6 in the protein backbone. Here, we set out to obtain a better understanding of the equilibrium mobility of GHSR by specifically labeling residue types in distinct regions of the molecule, addressing i) the transmembrane region, ii) the loops and helix ends, and iii) the C-terminus of the molecule. Measurement of motionally averaged dipolar couplings of the labeled amino acid types allowed the characterization of the dynamic properties of the regions in which these residues are predominantly located.

Solid-state NMR in general allows polarizing ^13^C spins either by CP from ^1^H nuclei or by direct polarization. While CP is generally preferred as it provides better signal due to excitation of highly sensitive hydrogens and shorter relaxation times of ^1^H, it is most efficient for rigid sites as they feature strongest dipolar couplings which are used for the polarization transfer. Therefore, CP-based excitation schemes contain a dynamic bias toward rigid structures (Schmidt et al., 2014; Thomas et al., 2015). Especially at a short CP contact time, only rigid protein segments are excited. Alternatively, in direct polarization, all ^13^C nuclei are excited equally and NMR experiments probing molecular dynamics using direct polarization provide an averaged response from both mobile and rigid sites. Here, we focus on discussing especially motional order parameters measured by CP using a very short contact time and direct excitation. Thus, rigid sites of the receptor and the dynamic signature of the entire molecule can be assessed individually.

The most straightforward assignment of the NMR signals of GHSR is the very intense signal from the C-terminal hexahistidine tag in the spectra of ^13^C-His labeled GHSR. Assignment was achieved on the basis of the high signal intensity and the coil like Cα/Cβ correlation peaks. Expectedly, we measured lowest order parameters of 0.17 ± 0.01 for the His tag at the C-terminal end using DIPSHIFT experiments with direct excitation, which corresponds to almost isotropic mobility.

In addition to the six His residues in the His tag, there are only three histidine residues in the sequence of GHSR, two of which in transmembrane helix 6 (His_258_ and His_280_) and one in the extracellular loop 2 (His_186_). If we assume that the former resonate with α-helical chemical shift and the latter with random coil chemical shift, we can assign these sites with some confidence. Using CP with a short contact time of 20 µs, we measure high order parameters of 0.80 ± 0.01 for the two α-helical residues in helix six likely representing His_258_ and His_280_, suggesting that they undergo only small amplitude fluctuations. His_186_ located in the second extracellular loop and likely featuring a random coil chemical shift exhibits a similar order parameter of 0.83 ± 0.02 suggesting that no motional difference exists between the transmembrane helices and the short loops of the receptor especially for extracellular loop 2 which is stabilized by the disulfide bridge. However, it is important to note that much lower order parameters are observed when DIPSHIFT experiments are conducted with longer CP contact time or directly excited, suggesting that individual GHSR molecules feature quite different dynamical properties.

Using ^13^C-Met-labeled GHSR, the central transmembrane segments were addressed. High order parameters of *S* = 0.85 ± 0.02 were found for the most rigid helical elements (as measured using CP with a short contact time), suggesting that these residues only undergo very small amplitude fluctuations. However, when all helical segments are excited by direct polarization, the order parameter for the α-helical sites decreases to 0.50 ± 0.03, suggesting larger motional amplitudes. Similar order parameters of 0.87 ± 0.03 to 0.81 ± 0.01 are obtained for the ^13^C-Arg residues at the flanking helix sites and in the loops connecting the helices. This is an important observation as it suggests that the loops of GHSR are indeed somewhat structured and dynamically stabilized. However, also the analysis of the mobility of all α-helical Met and Arg Cα sites excited by direct polarization confirm that GHSR is subject to heterogeneous dynamics as much lower order parameters between 0.50 ± 0.03 and 0.46 ± 0.04 are measured under these conditions. A recent study used DIPSHIFT experiments with CP excitation and also determined high order parameters between 0.7 and 0.8 for the Trp residues of the neuropeptide Y2 receptor, which also reside in rigid structures (Krug et al., 2020).

Also the side chain segments of the Met residues are relatively rigid, while the Arg feature somewhat higher mobility in spite of the fact that its guanidinium ion could be involved in salt bridges or hydrogen bonding. His side chains showed lowest order parameters. However, spectral resolution was not sufficient to distinguish between secondary structure elements rendering further conclusions rather speculative.

From the DIPSHIFT experiments no correlation times of the motions can be obtained, the experiments sample all motions with correlation times shorter than ∼40 µs. This suggests that the low order parameters measured for GHSR contain both segmental dynamics as well as reorientations of entire transmembrane helices.

Taken together, the segmental mobility of GHSR determined for α-helices (as reflected in the order parameters of the Met) and for coil segments (as indicated in the order parameters of the Arg) is roughly comparable to the order parameters determined for fully-labelled GHSR (Schrottke et al., 2017). In contrast, the order parameters of His in the coil regions were significantly lower after direct excitation and with a CP contact time of 700 µs indicating the expected higher mobility of the C-terminus.

In the context of other membrane proteins, our analysis of the molecular dynamics of GHSR suggests that i) the molecular dynamics of GHSR is heterogeneous and ii) the molecule is significantly more mobile than other membrane proteins of similar size as reflected in the lower order parameters. For *Anabena* sensory rhodopsin having similar secondary structure order parameters in the range between 0.9 and 1.0 were determined from NMR relaxation experiments (Good et al., 2014). The α-helical membrane protein colicin Ia showed order parameters between 0.88 and 0.93 (Huster et al., 2001) and residues within the β-barrel of KpOmpA exhibited average order parameters of 0.88 (Saurel et al., 2017). In contrast to these highly rigid membrane proteins GPCRs and GHSR in particular undergo fast transitions between different conformational states and fluctuations of varying amplitudes. These include not only segmental motions but also motions of the α-helices with respect to each other. Some contribution to the low order parameters may come from the axially symmetric reorientation of the receptor in the membrane as observed for the CXCR1 (Park et al., 2011) but also segmental mobility and fast reorientations of the α-helices in the liquid-crystalline membranes have to be considered. Furthermore, the constitutive activity that keeps the receptor to a certain degree in the activated state might contribute to its dynamic properties.

## Conclusion

A simple and convenient protocol was established to prepare the GHSR by CF expression as precipitate with subsequent refolding into a functional form and membrane reconstitution. The recorded NMR spectra suggest that about 80% of the receptor was reconstituted in a functional form. Amino acid type-specific ^13^C labeling was achieved in the CF synthesis allowing simplification of the solid-state NMR spectra of the receptor molecules reconstituted into DMPC membranes. Without having obtained site-specific assignment of the NMR signals, it was still possible to obtain dynamics information of specific regions of the receptor allowing the description of the dynamics of specific segments of GHSR. While the GHSR is overall a very dynamic molecule, it was confirmed that the membrane embedded seven α-helices constitute the most rigid part of the molecule. Also, the loop structures connecting the transmembrane segments show a similar rigidity as the transmembrane segments. Expectedly, the C-terminus of the molecule represents the most flexible part undergoing almost isotropic motions. The mobility of GHSR is heterogeneous. If an NMR excitation scheme is used that only polarizes highly rigid receptor segments, only receptors undergoing small amplitude fluctuations are detected. However, when all ^13^C nuclei are polarized equally, the motional amplitudes are much larger suggesting substantial segmental mobility as well as fluctuations of entire secondary structure elements. The work presented here paves the way to study single residues and their dynamic properties as a function of ligand binding, constitutive activity, and recruitment of intracellular binding partners. However, site-specific detection of the NMR signals is a prerequisite for such experiments. This will require more elaborate preparative procedures as well as increased resolution and sensitivity of the NMR spectra. Despite the necessity for site-selective labeling and assignment, studies along these lines will reveal more molecular details required to fully understand the interesting biology of GPCR activation and function on the basis of their structure and dynamics.

## Data Availability Statement

The raw data supporting the conclusions of this manuscript will be made available by the authors, without undue reservation, to any qualified researcher.

## Author Contributions

EMP prepared the samples including CF expression and reconstitution, performed and evaluated NMR experiments, and contributed figures and text elements to the manuscript; Ulrike Krug. and FS prepared the DNA constructs and optimized CF expression; HAS supported and supervised NMR measurements; FB provided expert knowledge and support on CF expression; PS and DH supervised the data evaluation and interpretation; Ulrike Krug and DH supervised the project and wrote the manuscript with the contributions from all coauthors.

## Funding

The project was funded by a junior research grant by the Medical Faculty (Leipzig University) assigned to Ulrike Krug and from the Deutsche Forschungsgemeinschaft (DFG, German Research Foundation) through CRC 1423, project number 421152132, subproject A02.

## Conflict of Interest

The authors declare that the research was conducted in the absence of any commercial or financial relationships that could be construed as a potential conflict of interest.

## Abbreviations

**Abbreviations:** CF, cell-free; CP, cross polarization; DHPC, 1,2-diheptanoyl-*sn*-glycero-3-phosphocholine; DIPSHIFT, dipolar and chemical shift correlation; DMPC, 1,2-dimyristol-*sn*-glycerol-3-phosphocholine; GHSR, growth hormone secretagogue receptor; GPCR, G protein-coupled receptor; MAS, magic angle spinning; NMR, nuclear magnetic resonance.
