# Supplementary material for: Integration of Cell-Free Expression and Solid-State NMR to Investigate the Dynamic Properties of Different Sites of the Growth Hormone Secretagogue Receptor
Source: Front Pharmacol. 2020 Oct 29;11:562113. doi: 10.3389/fphar.2020.562113 (PMC7723455; doi:10.3389/fphar.2020.562113)
Supplement: Supplementary file 1 [file DataSheet1_v1.PDF]

Supplementary Material

Integration of Cell-Free Expression and Solid-State NMR to Investigate the Dynamic Properties of Different Sites of the Growth Hormone Secretagogue Receptor

Emelyne M. Pacull,[1] Franziska Sendker,[1] Frank Bernhard,[2,3] Holger A. Scheidt,[1] Peter Schmidt,[1] Daniel Huster,[1,*] Ulrike Krug[1,*]

[1] Institute for Medical Physics and Biophysics, University of Leipzig, Leipzig, Germany.

[2] Institute of Biophysical Chemistry, Goethe University Frankfurt, Frankfurt am Main, Germany.

[3] Center for Biomolecular Magnetic Resonance, Goethe University Frankfurt, Frankfurt am Main, Germany.

1 Supplementary Figures and Tables

1.1 Supplementary Figures

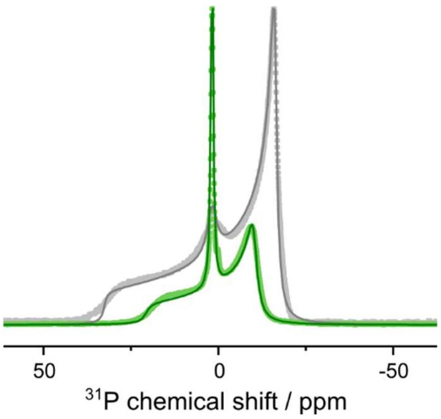

Supplementary Figure S1: Static ^{31}P NMR spectra of isotopically ^{13}C-His labeled GHSR reconstituted in DMPC-*d*$_{54}$ membranes (green) and of DMPC membranes without receptors (gray) determined at 37°C. The spectra exhibit the typical line shape of a liquid-crystalline membrane bilayer. The received spectra were simulated (solid lines) using a program written in Mathcad to obtain the chemical shift anisotropy (CSA). In the presence of the receptor, the CSA of the axial symmetric part decreased from 49 ppm (without receptor) to 38 ppm while the isotropic contribution to the spectrum is increased from about 5% to 19%.

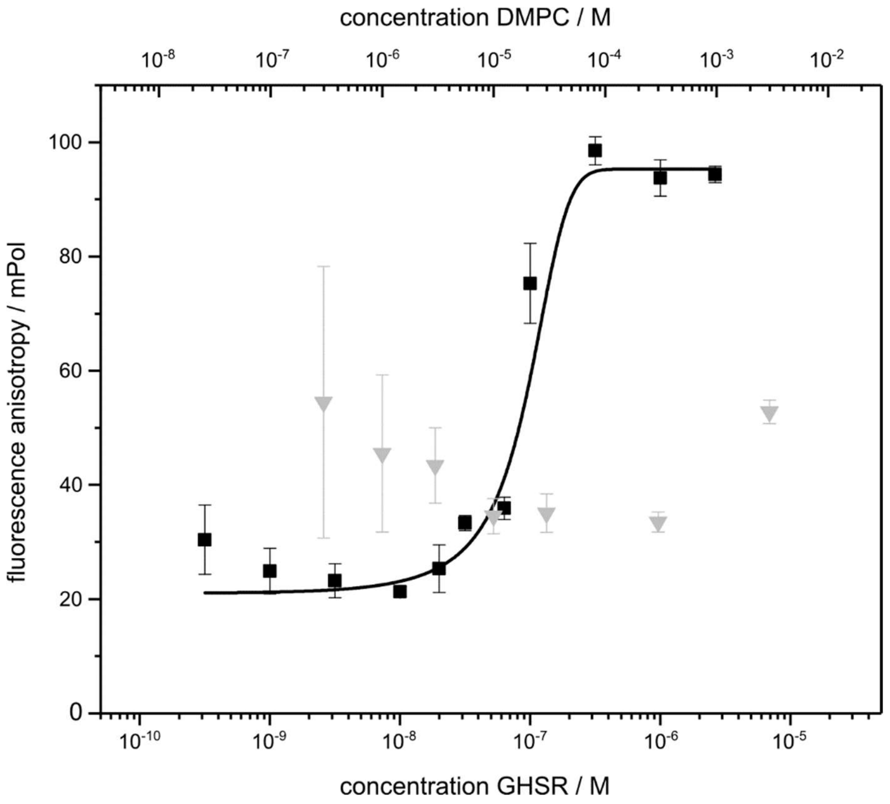

Supplementary Figure S2. Fluorescence polarization assay to monitor ligand binding to GHSR prepared by CF expression (black symbols). Varying concentrations of GHSR were incubated with 50 nM ATTO520-ghrelin. The average of the EC_{50} value of three independent experiments (each in duplicate or in triplicate) was determined to be 56 ± 30 nM by fitting the data with a sigmoidal dose-response curve using the Origin Software (solid line). The gray data points represent binding of ATTO520-ghrelin to DMPC membranes in the absence of the GHSR. The x-axis on top indicates the DMPC concentrations corresponding to the receptor concentrations given on the bottom x-axis.

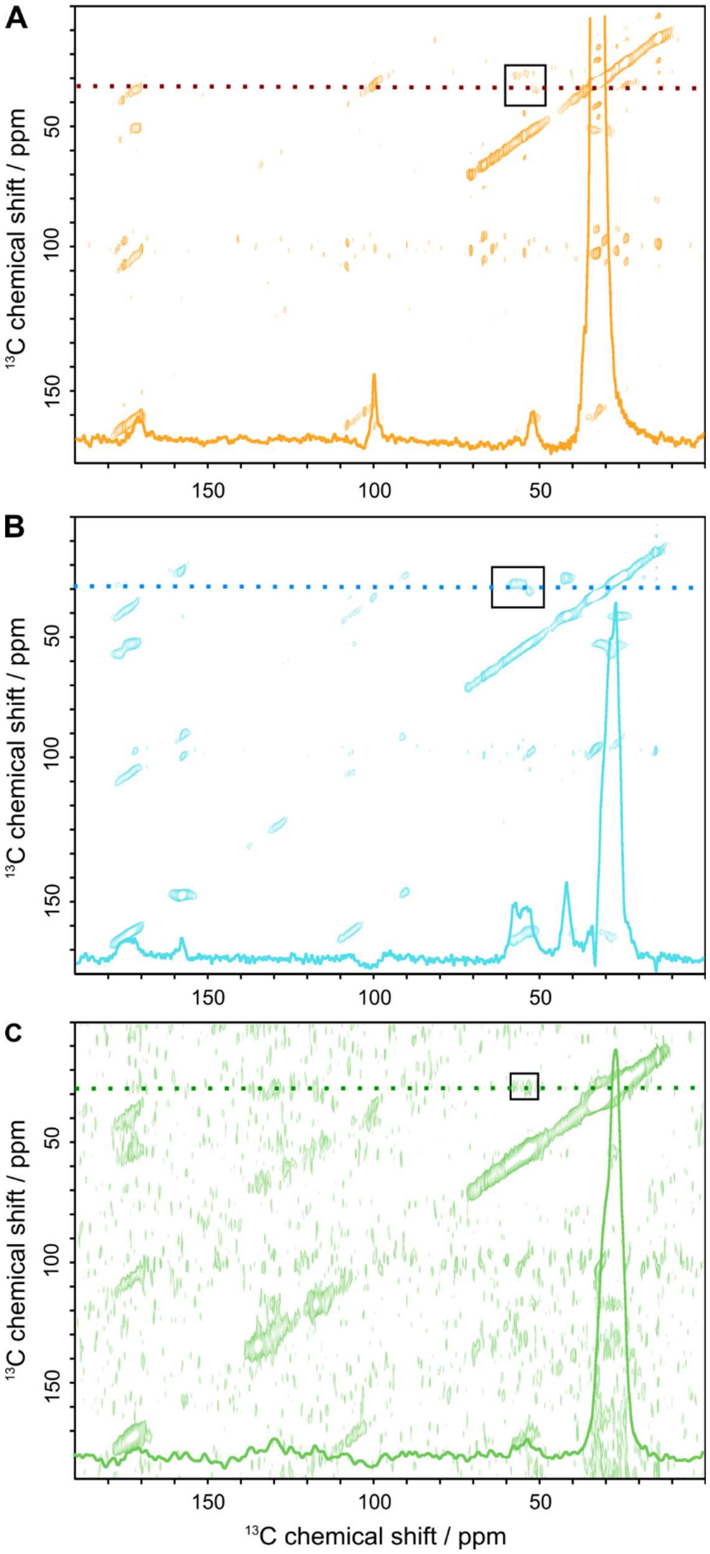

Supplementary Figure S3: Full ^{13}C-^{13}C DARR NMR spectra and 1D cross sections of isotopically labeled GHSR in DMPC membranes. Experiments were carried out at -30°C and an MAS rate of 11,777 Hz. Solid boxes indicate the spectral area corresponding to the Cα/Cβ correlations in Fig. 3 of the main text. GHSR was labeled with (A) ^{13}C-Met (orange), (B) ^{13}C-Arg (blue), and (C) ^{13}C-His (green). Solid lines represent 1D cross sections at (A) 33.4 ppm, (B) 28.4 ppm, and (C) 27.4 ppm (dotted lines). The signal-to-noise ratio for the Cα/Cβ crosspeaks was determined to (A) 10.8, (B) 11.5, and (C) 5.3.

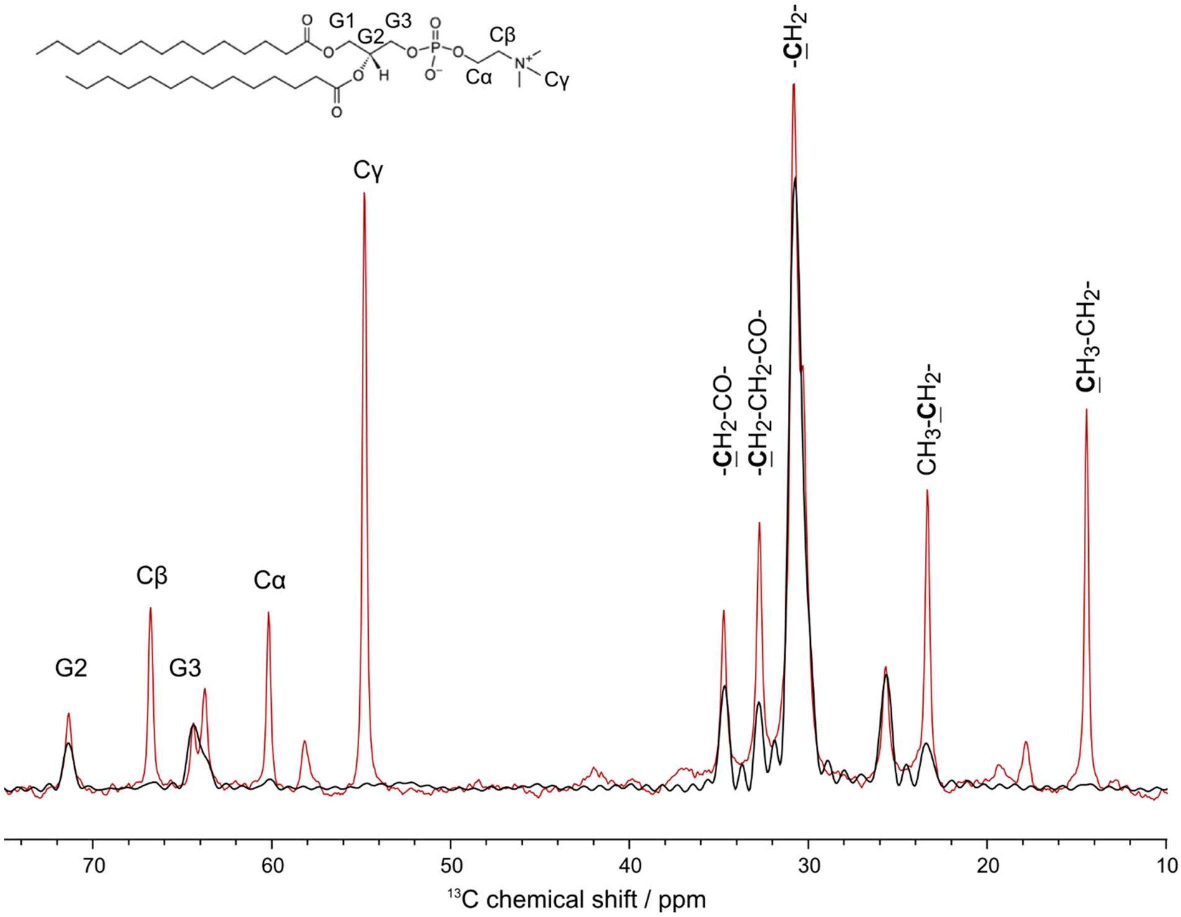

Supplementary Figure S4: ^{13}C MAS NMR spectra of DMPC membrane at 37°C and a MAS rate of 11,777 Hz. NMR spectra were acquired using a CP contact time of 700 µs (black) or direct excitation (red). Signals were assigned to the carbon atoms of DMPC of which the structure is given.

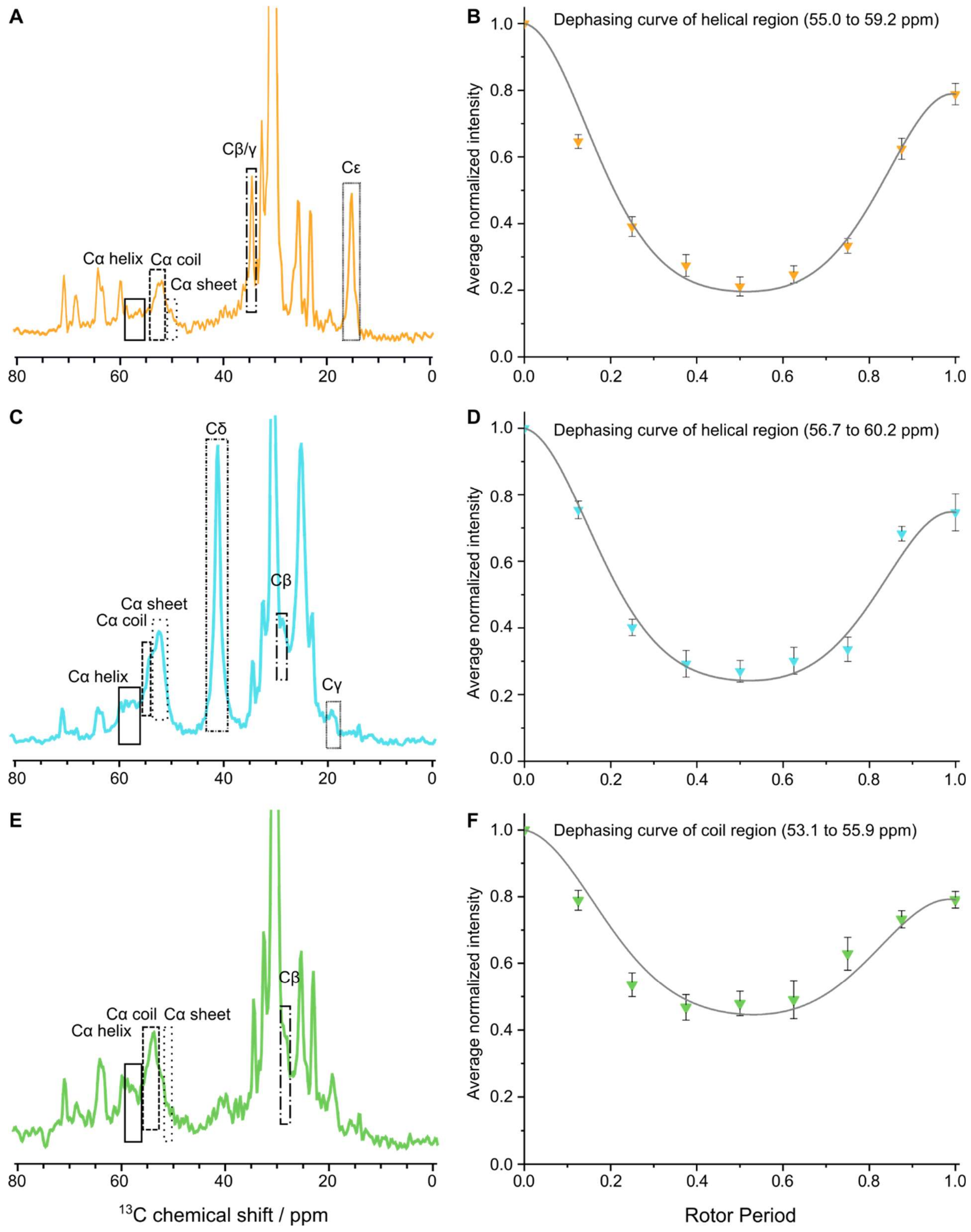
A
Cα helix
Cα coil
Cα sheet
Cβ/γ
Cε
B
Dephasing curve of helical region (55.0 to 59.2 ppm)
C
Cα helix
Cα coil
Cα sheet
Cδ
Cβ
Cγ
D
Dephasing curve of helical region (56.7 to 60.2 ppm)
E
Cα helix
Cα coil
Cα sheet
Cβ
F
Dephasing curve of coil region (53.1 to 55.9 ppm)
Average normalized intensity
13C chemical shift / ppm
Rotor Period

Supplementary Figure S5: Experimental details of the DIPSHIFT NMR experiments on GHSR reconstituted in DMPC membranes. ^{13}C-Met (orange, (A), (B)), ^{13}C-Arg (blue, (C), (D)), and ^{13}C-His (green, (E), (F)) labeled GHSR samples were investigated. (A), (C), and (E) show exemplified first increments of ^{1}H-^{13}C DIPSHIFT NMR spectra measured at 37°C and an MAS rate of 5000 Hz with a CP contact time of 700 μs. Spectral regions were assigned to α-helical, random coil, and β-sheet secondary structures of the backbone atoms as well as the side chain atoms. The dephasing curves shown in (B), (D), and (F) represent the average of four independent sample preparations and were used to calculate the order parameters. The error bars given for the data points of the dephasing curves were calculated as the standard error of the mean.

1.2 Supplementary Tables

Supplementary Table S1: Additives to the fermentation medium for growth of *E. coli* Rosetta cells used for preparation of the S12 extract.

Chemicals	Supplier	CAS number	Molecular weight (g/mol)	Amount per 1 l medium
Choline chloride	Sigma-Aldrich	67-48-1	139.62	28.6 mg
Niacin (Nicotinic acid)	Roth	59-67-6	123.11	25.1 mg
p-Aminobenzoic acid	Sigma-Aldrich	150-13-0	137.14	25.6 mg
Pyridoxine hydrochloride	Roth	58-56-0	205.6	1.5 mg
Riboflavin	Roth	83-88-5	376.37	3.9 mg
Thiamin hydrochloride	Roth	67-03-8	337.27	17.7 mg
Betaine	Sigma-Aldrich	107-43-7	117.15	33.1 mg
Biotin	Roth	58-85-5	244.31	0.1 mg
Folinic acid calcium salt hydrate	Sigma-Aldrich	1492-18-8	511.5	0.075 mg
$FeCl_3*6\ H_2O$	Fluka	10025-77-1	270.3	20 mg
$Na_2MoO_4*2\ H_2O$	Roth	10102-40-6	241.95	3.5 mg
Boric acid	Roth	10043-35-3	61.84	1.2 mg
$CoCl_2*6\ H_2O$	Roth	7791-13-1	237.93	3.4 mg
$CuSO_4*5\ H_2O$	riedel-de haen	7758-99-8	159.61	2.175 mg
$MnSO_4*H_2O$	Sigma-Aldrich	10034-96-5	169.02	1.9 mg
$ZnSO_4*7\ H_2O$	Fluka	7446-20-0	287.56	3.4 mg
L-Aspartic acid sodium salt monohydrate	Fluka	323194-76-9	173.11	28.5 mg
Glycine	Roth	56-40-6	75.07	49.1 mg
L-Histidine	Roth	71-00-1	155.15	9.3 mg
L-Isoleucine	Sigma-Aldrich	73-32-5	131.17	26.2 mg
L-Leucine	Sigma-Aldrich	61-90-5	131.2	29.9 mg
L-Lysine monohydrochloride	Sigma-Aldrich	657-27-2	182.65	43.1 mg
L-Methionine	Sigma-Aldrich	63-68-3	149.21	14.9 mg
L-Phenylalanine	Sigma-Aldrich	63-91-2	165.19	15.3 mg
L-Proline	Sigma-Aldrich	147-85-3	115.13	31.8 mg
L-Threonine	Sigma-Aldrich	72-19-5	119.12	37.7 mg
L-Tryptophane	Sigma-Aldrich	73-22-3	204.23	102.1 mg
L-Tyrosine	Sigma-Aldrich	60-18-4	181.19	15.6 mg
L-Valine	Sigma-Aldrich	72-18-4	117.15	117.1 mg

Supplementary Table S2: Exemplified table for pipetting a 1 ml CF reaction to express GHSR. Given are the concentrations of the stocks, the final concentrations as well as the volumes to be pipetted. The master mix (MM) is split in a ratio of 16.05 and 0.95 to be added to the feeding mix (FM) and the reaction mix (RM), respectively.

Compound	***Concentrations***		***Volume***		
	**Stock**	**Final**	**MM**	**FM**	**RM**
ddH$_2$O			491 µl		
HEPES buffer (pH 8.4)	2.5 M	92 mM	660 µl		
Mg(OAc)$_2$	2.0 M	8.4 mM	75.2 µl		
KOAc	4.0 M	106 mM	476 µl		
PEG 8000	40 % (w/v)	2 % (w/v)	900 µl		
NaN$_3$	10 % (w/v)	0.05 % (w/v)	90 µl		
Folic acid	20 mg/ml	0.1 mg/ml	90 µl		
DTT	500 mM	2 mM	72 µl		
NTP	75×	1×	240 µl		
cOmplete (Protease Inhibitor cocktail)	50×	1×	360 µl		
Phosphoenolpyruvate-KOH (PEP)	1.0 M	20 mM	360 µl		
Lithium Potassium Acetyl Phosphate (ACP)	1.0 M	20 mM	360 µl		
<u>amino acid mix</u>					
amino acid mix w/o L-Cys, w/o labeled aa	4.35 mM	0.97 mM	2013 µl		
L-Cys	100 mM	0.97 mM	87.5 µl		
labeled amino acid (L-Met, L-Arg, or L-His)	100 mM	0.97 mM	87.5 µl		
<u>RCWMDE mix</u>					
RDE	33 mM	1 mM	538 µl		
L-Cys	100 mM	1 mM	180 µl		
L-Trp	50 mM	1 mM	180 µl		
L-Met or labeled L-Met	100 mM	1 mM	180 µL		
<u>AFSLTV mix</u>	16.7 mM	1 mM	1078 µl		
**Total volume MM**			**8518 µl**		
Split MM into FM + RM (ratio 16.05 : 0.95)				8042 µl	476 µl
<u>amino acid mix</u>					
amino acid mix w/o L-Cys, w/o labeled aa	4.35 mM	0.97 mM		2013 µl	
L-Cys	100 mM	0.97 mM		87.5 µl	
labeled amino acid (L-Met, L-Arg, or L-His)	100 mM	0.97 mM		87.5 µl	
S30 C buffer	100 %	35 %		5950 µl	
Pyruvate kinase	10 mg/ml	0.04 mg/ml			4.0 µl
tRNA	40 mg/ml	0.5 mg/ml			12.5 µl
T7 RNA Polymerase (if not in S30 extract)	200 U/µl	6 U/µl			30.0 µl
Ribolock RNAse Inhibitor	40 U/µl	0.3 U/µl			7.5 µl
DNA (SER-GHSR×pIVEX2.3d)	1204 µg/ml	26 µg/ml			21.6 µl
S30 extract	100 %	40 %			400 µl
ddH$_2$O				820.4 µl	48.4 µl
**Total (µL)**				**17000** µl	**1000** µl
		**pipetting steps**	**1st**	**2nd**	**3rd**
			the total volume of MM was split in a ratio of 16.05 (FM) : 0.95 (RM) and the reagents indicated for FM and RM were added		

Supplementary Table S3: Order parameters of DMPC signals determined in DIPSHIFT experiments at different CP contact times and from a directly excited spectrum.

DIPSHIFT experiment	55.4-54.4 ppm	35.2-34.5 ppm	33.2-32.2 ppm	31.2-30.2 ppm	26.2-25.2 ppm	23.8-22.8 ppm
	**Cγ**	-**C**H_2-CO-	-**C**H_2-CH_2-CO-	-**C**H_2-		CH_3-**C**H_2-
CP 20 µs	n.d. †	0.37	0.16	0.13	0.10	0.36
CP 700 µs	0.31	0.19	0.17	0.21	0.22	0.10
CP 2000 µs	0.19	0.19	0.17	0.22	0.23	0.14
direct	0.04	0.21	0.16	0.20	0.19	0.12

† n.d.: not determined due to insufficient spectral intensity

Supplementary Table S4: Order parameters of backbone Cα atoms of isotopically labeled GHSR reconstituted into DMPC bicelles at a temperature of 37°C obtained from DIPSHIFT experiments with varying CP contact times or by direct excitation. The table reports the chemical shift regions integrated to obtain order parameters and the deuteration scheme of the DMPC membrane. The secondary structure assignment is based on the Biological Magnetic Resonance Bank (BMRB).

^{13}C-Met GHSR	DIPSHIFT excitation scheme	59.2-58.2 ppm	58.2-57.4 ppm	57.4-56.8 ppm	56.8-56 ppm	56-55 ppm	54.8-53.8 ppm	53.7-52.7 ppm	52.7-51.9 ppm
Membrane system		*helix*	*helix*	*helix*	*helix*	*helix*	*coil*	*coil*	*sheet*
DMPC-d_{54}	CP 20 µs	0.95	0.86	0.81	0.86	0.83	0.78	0.86	0.89
DMPC-d_{54}	CP 20 µs	0.87	0.86	0.89	0.88	0.94	0.81	0.88	0.91
DMPC-d_{13}	CP 20 µs	† n.d.	† n.d.	† n.d.	1.03	0.80	0.94	0.77	0.88
DMPC	CP 20 µs	0.74	0.84	0.80	0.79	0.72	† n.d.	0.82	0.96
DMPC-d_{54}	CP 700 µs	0.72	0.82	0.90	0.86	0.81	0.75	0.79	0.84
DMPC-d_{54}	CP 700 µs	0.71	0.82	0.83	0.82	0.91	0.82	0.84	0.87
DMPC-d_{13}	CP 700 µs	0.79	0.70	0.71	0.74	0.79	0.84	0.76	0.80
DMPC	CP 700 µs	0.67	0.70	0.73	0.78	0.70	0.60	0.66	0.78
DMPC-d_{54}	CP 2000 µs	0.83	0.84	0.82	0.80	0.80	0.77	0.84	0.86
DMPC-d_{13}	CP 2000 µs	0.83	0.90	0.89	0.76	0.78	0.86	0.77	0.83
DMPC	CP 2000 µs	0.51	0.67	0.63	0.79	0.68	0.54	0.57	0.74
DMPC-d_{54}	direct	0.55	0.54	0.62	0.60	0.48	0.25	0.49	0.69
DMPC-d_{13}	direct	0.37	0.27	0.35	0.71	0.54	0.28	0.37	0.67
DMPC	direct	0.37	0.52	0.51	0.52	0.50	0.20	0.43	0.65

^{13}C-Arg GHSR	DIPSHIFT excitation scheme	60.2-59.2 ppm	59.1-58.1 ppm	58.1-57.3 ppm	57.3-56.7 ppm	56.6-55.6 ppm	55.5-54.5 ppm	53.3-52.3 ppm	
Membrane system		*helix*	*helix*	*helix*	*helix*	*coil*	*coil*	*sheet*	
DMPC-d_{54}	CP 20 µs	0.74	0.89	0.89	0.82	0.84	0.86	0.91	
DMPC-d_{54}	CP 20 µs	1.12	† n.d.	1.09	0.89	0.81	0.80	0.96	
DMPC-d_{13}	CP 20 µs	1.02	0.85	0.74	0.81	0.79	0.76	0.89	
DMPC	CP 20 µs	0.66	0.77	0.82	0.87	0.82	0.78	0.83	
DMPC-d_{54}	CP 700 µs	0.70	0.87	0.87	0.85	0.80	0.73	0.81	
DMPC-d_{54}	CP 700 µs	0.52	0.74	0.73	0.72	0.69	0.63	0.76	
DMPC-d_{13}	CP 700 µs	N/A	0.74	0.86	0.67	0.77	0.65	0.78	
DMPC	CP 700 µs	0.55	0.72	0.71	0.69	0.66	0.59	0.74	
DMPC-d_{54}	CP 2000 µs	0.41	0.73	0.74	0.69	0.71	0.64	0.77	
DMPC-d_{13}	CP 2000 µs	0.77	0.69	0.66	0.76	0.73	0.62	0.87	
DMPC	CP 2000 µs	0.47	0.81	0.77	0.72	0.71	0.61	0.78	
DMPC-d_{54}	direct	0.22	0.29	0.25	0.46	0.55	0.28	0.58	
DMPC-d_{13}	direct	0.46	0.48	0.55	0.42	0.58	0.47	0.64	
DMPC	direct	0.45	0.66	0.51	0.52	0.55	0.41	0.62	

**^{13}C-His GHSR**	DIPSHIFT excitation scheme	58.3-57.3 ppm	57.3-56.3 ppm	55.9-55.5 ppm	55.5-55.1 ppm	54.5-53.7 ppm	53.7-53.1 ppm	51.7-50.7 ppm	
Membrane system		helix	helix	coil	coil	coil	coil	sheet	
DMPC-d_{54}	CP 20 µs	0.76	0.81	0.91	0.84	0.74	† n.d.	† n.d.	
DMPC-d_{54}	CP 20 µs	† n.d.	† n.d.	0.79	0.80	0.91	0.91	0.89	
DMPC-d_{13}	CP 20 µs	† n.d.	† n.d.	0.61	0.80	0.85	0.88	0.83	
DMPC	CP 20 µs	0.81	0.81	0.91	0.83	0.79	0.91	0.81	
DMPC-d_{54}	CP 700 µs	0.71	0.70	0.66	0.56	0.50	0.44	0.74	
DMPC-d_{54}	CP 700 µs	0.80	0.72	0.69	0.58	0.55	0.63	0.68	
DMPC-d_{13}	CP 700 µs	0.89	0.77	0.68	0.53	0.48	0.57	0.82	
DMPC	CP 700 µs	0.79	0.75	0.73	0.70	0.48	0.55	0.68	
DMPC-d_{54}	CP 2000 µs	0.71	0.69	0.76	0.71	0.45	0.50	0.76	
DMPC-d_{54}	CP 2000 µs	0.69	0.83	0.60	0.51	0.46	0.44	0.62	
DMPC-d_{13}	CP 2000 µs	0.99	0.91	0.63	0.55	0.49	0.38	0.68	
DMPC	CP 2000 µs	0.77	0.76	0.74	0.59	0.38	0.39	0.75	
DMPC-d_{54}	direct	0.45	0.35	0.26	0.16	0.09	0.15	0.62	
DMPC-d_{54}	direct	0.51	0.50	† n.d	0.14	0.17	0.18	0.41	
DMPC-d_{13}	direct	0.22	0.41	0.38	0.29	0.19	0.18	0.64	
DMPC	direct	0.39	0.41	0.26	0.16	0.18	0.18	0.40	

† n.d.: not determined due to insufficient spectral intensity

Supplementary Table S5: Order parameters of side chain carbon atoms from ^{13}C-Met, ^{13}C-Arg, and ^{13}C-His labeled GHSR at a temperature of 37°C obtained from DIPSHIFT experiments with different CP contact times or by direct excitation. According to chemical shift tables reported in the BMRB, the NMR signals were assigned to the side chain. Side chain peaks that might have been influenced by lipid signals were not considered in the analysis.

**^{13}C-Met GHSR**	DIPSHIFT experiment	36.4-35.2 ppm	15.8-14.8 ppm	
		Cβ / Cγ	Cε	
DMPC-d_{54}	CP 20 µs	0.72	† n.d	
DMPC-d_{54}	CP 20 µs	0.73	† n.d	
DMPC-d_{54}	CP 700 µs	0.60	0.15	
DMPC-d_{54}	CP 700 µs	0.70	0.18	
DMPC-d_{54}	CP 2000 µs	0.64	0.14	
DMPC-d_{54}	direct	0.60	0.11	
**^{13}C-Arg GHSR**	DIPSHIFT experiment	41.9-40.9 ppm	29.0-28.0 ppm	19.7-18.7 ppm
		Cδ	Cβ	Cγ
DMPC-d_{54}	CP 20 µs	0.61	0.46	0.48
DMPC-d_{54}	CP 20 µs	0.50	0.42	0.44
DMPC-d_{54}	CP 700 µs	0.40	0.44	0.36
DMPC-d_{54}	CP 700 µs	0.33	0.37	0.37
DMPC-d_{54}	CP 2000 µs	0.29	0.37	0.40
DMPC-d_{54}	direct	0.22	0.21	0.32
**^{13}C-His GHSR**	DIPSHIFT experiment	28.8-27.8 ppm		
		Cβ		
DMPC-d_{54}	CP 20 µs	0.38		
DMPC-d_{54}	CP 20 µs	0.46		
DMPC-d_{54}	CP 700 µs	0.31		
DMPC-d_{54}	CP 700 µs	0.41		
DMPC-d_{54}	CP 2000 µs	0.34		
DMPC-d_{54}	CP 2000 µs	0.17		
DMPC-d_{54}	direct	0.11		
DMPC-d_{54}	direct	0.14		
